# Kangaroo Care for Relieving Neonatal Pain Caused by Invasive Procedures: A Systematic Review and Meta-Analysis

**DOI:** 10.1155/2022/2577158

**Published:** 2022-09-23

**Authors:** Yunan Zhao, Yanjun Dong, Jie Cao

**Affiliations:** Department of Neonatology, The First Hospital of China Medical University, Shenyang, Liaoning 110001, China

## Abstract

**Objective:**

Neonates develop significant pain responses during invasive procedures, and nonpharmacological interventions are better means of pain relief. An increasing number of studies have confirmed the effectiveness of kangaroo care (KC) in relieving neonatal pain caused by invasive procedures, but conclusions are inconsistent. In this study, a literature search and meta-analysis were performed to evaluate the effect of kangaroo care on relieving neonatal pain.

**Methods:**

The works of literature related to the application of KC in neonatal invasive procedures in the databases of Pubmed, Embase, Springer Link, Ovid, CNKI, and CBM were searched, and the RCT literature from database establishment to July 2022, was selected to evaluate the risk of bias, combined with statistical pain relief outcome indicators.

**Results:**

12 pieces of literature were finally included in this study, with a total of 1172 newborns, including 585 newborns (49.9%) using KC and 587 newborns (50.1%) using the control group method. Meta-analysis showed that an infant's heart rate during invasive procedures under KC intervention was significantly lower than that of other interventions (*MD* = −6.77, 95% CI (−13.03, −0.50), *Z* = −2.12, *P*=0.03), but compared to other nonpharmacological interventions, there was no clear advantage in the overall evaluation of pain reduction in infants (*MD* = −0.36, 95% CI (−0.80, 0.08), *Z* = −1.60, *P*=0.11).

**Conclusion:**

The heart rate of KC intervention during invasive procedures in infants is significantly lower than that of other interventions, and it can significantly relieve pain in infants, but the effect is not more than that of oral sucrose (or glucose) or standard care. KC combined with oral sucrose may achieve a better pain relief effect in infants, but more studies are still needed to verify it.

## 1. Introduction

Compared with older children and adults, newborns are more sensitive, intense, diffuse, and persistent in the perception of pain, and pain can cause significant physiological reactions, allergic reactions, and even chronic pain syndrome, and long-term physical discomfort, and lead to a series of short-term and long-term adverse effects such as difficulty concentrating, anxiety, cognitive behavioral disorders, poor adaptability, and growth retardation in childhood [1–3]. Neonatal pain is mainly derived from various invasive procedures during hospitalization (including heel blood sampling, arteriovenous puncture, intramuscular injection, and lumbar puncture) [4]. Different from adults, studies have reported that 80% to 90% of analgesic drugs have different degrees of adverse reactions in newborns [5], so it is difficult to widely use drugs to intervene in neonatal pain in clinical practice. At present, there are more nonpharmacological intervention methods to relieve the symptoms of newborns, such as nonnutritive sucking, oral sucrose (or glucose), etc. [6]. Kangaroo care (KC), also known as skin-to-skin care, aims to provide humanized care for newborns, reduce the separation time between children and parents, promote closer mother-child relationships, make parents more confident in coping with low birth weight, and continuously improve the physical and behavioral stability of infants [7]. Increasing studies have confirmed the effectiveness of KC in relieving neonatal pain caused by invasive procedures, and compared with drug intervention, it has the characteristics of less risk, simplicity, and economic applicability. Although many literature studies on relieving neonatal pain caused by invasive procedures through KC have emerged at home and abroad in recent years, the conclusions obtained are not completely consistent due to insufficient sample size, poor systematicness and integrity of design, and different results in some single-randomized controlled trials (RCTs). In a study by Campbell-Yeo et al. [8], 81 neonates randomized to KC were compared with 81 neonates treated with oral sucrose, and the results showed that there was no significant difference in the neonatal pain score (PIPP, premature infant pain profile score) after heel blood sampling compared with the control group treated with oral sucrose. However, in another randomized controlled study by de Sousa Freire et al. [9], newborns who also received KC intervention had lower PIPP scores after heel prick compared with newborns who received sucrose intervention, and the difference was statistically significant. The conclusions of such studies are completely different, and it is difficult to safely and effectively use kangaroo care to guide the clinical relief of neonatal pain. Therefore, this study aims to combine and analyze the relevant published literature studies by meta-analysis, in order to objectively evaluate the effect of KC on relieving neonatal pain and provide strong empirical evidence for the management of clinical neonatal pain.

## 2. Materials and Methods

### 2.1. Literature Database

In this study, Pubmed, Embase, Springer Link, and Ovid were selected as English database sources, CNKI and CBM were selected as Chinese database sources, and the publication time of these pieces of literature was from the beginning of database establishment to July 2022. Two colleagues searched the pieces of literature published in the database with the keywords (“*Kangaroo nursing*” OR “*Kangaroo care*” OR “*skin to skin care*”) AND (“*premature infant*”) AND (“*pain*”) AND (“*RCT*” OR “*Randomized controlled trial*”). Generally speaking, keywords with similar significance were connected with “*OR*,” while those without similarity were connected with “*AND*.”

### 2.2. Literature Inclusion and Exclusion Criteria

#### 2.2.1. Inclusion Criteria

The inclusion criteria were as follows: ① All pieces of literatures were single-center or multicenter-randomized controlled trials (*RCTs*); ② all subjects were infants born within one month, including full-term infants or premature infants, during hospitalization, the child underwent invasive procedures due to screening, diagnosis, and treatment of diseases (such as heel blood sampling, venipuncture, and intramuscular injection); ③ all studies were divided into the experimental group and the control group according to the randomization method for intervention, and we will perform a quality evaluation for the randomization process of each study. Children in the experimental group were intervened with kangaroo care (or skin-to-skin care) when undergoing invasive procedures, and infants were held in a comfortable position by their parents (specific measures varied depending on each study); in the control group, standard care or minimal care or other intervention methods (such as oral sucrose/glucose) were used [10].

#### 2.2.2. Exclusion Criteria

The exclusion criteria were as follows: ① we excluded non-RCT studies (such as controlled clinical studies, observational studies, and review studies); ② studies containing analgesics or sedatives within 24 to 72 hours before invasive procedures, or critically ill neonates unresponsive to painful stimuli; ③ studies which were unable to obtain outcome indicators or unable to obtain data on outcome indicators; ④ combined interventions, such as KC combined with sucrose; ⑤ studies that did not compare KC with other interventions for the purpose of the study, such as the comparison of the effect of KC performed by the father and mother; ⑥ literature studies in which the implementation scenario is specified as invasive operation in the study, such as the literature in the study in which the implementation scenario is lactation.

### 2.3. Literature Quality Evaluation

Cochrane risk of bias V2.0 [11] was used to perform a quality evaluation for the included randomized controlled studies. This standard includes 5 domains (randomization process, implementation bias, data bias, data measurement bias, and selection bias) and 1 overall bias assessment. “Low risk,” “some concerns of risk,” and “high risk” were used to evaluate each domain (or overall bias).

### 2.4. Outcome Indicators

Primary outcome indicators included the following indicators: (a) premature infant pain profile score (PIPP) [12]; (b) infant heart rate at manipulation; (c) infant oxygen saturation at manipulation. Although there are many scales to measure pain in newborns, we only take the most applied one, that is, the PIPP scale, which scores infant's responses to invasive procedures from fetal age, behavioral status, highest heart rate, oxygen desaturation, frowning, squeezing eyes, and deepening nasolabial folds, with higher scores indicating higher pain. The measurement time points are 2 to 5 min after invasive response, if the measurement data of multiple time points are reported in the literature, only the data of 2 min will prevail. If too few articles were included, we only performed descriptive analysis for indicators.

### 2.5. Literature Screening

The pieces of literature retrieved from the database were screened by two colleagues, after the software deduplication, the abstract part was read, and the remaining pieces of literature were obtained after preliminary screening according to the previously established inclusion and exclusion criteria after full text reading, and finally, after quality evaluation, the pieces of literature with serious bias and low quality were removed, and the studies were finally included in the analysis.

### 2.6. Data Extraction

Data extraction was performed by the researcher by reading the full text, and data such as interventions, the total number of people, grouping, characteristics of study subjects, and outcome indicators were extracted and entered into Excel sheets.

### 2.7. Statistical Methods

(a) Continuous data (PIPP score, HR, and SPO2) used pooled MD with 95% CI as effect size and as the random-effect model, *P* < 0.05 was considered statistically significant, and the combined results were presented on forest plots; (b) for literature heterogeneity, Tau values were calculated, using *Q* check, and *P* < 0.05 was used to indicate heterogeneity of the results; (c) if there was heterogeneity between literature studies, subgroup analysis was adopted to assess the impact factor, and impact analysis was used to diagnose the stability of the results; (d) publication bias was quantified using Egger's test and presented using contour-enhanced funnel plots.

## 3. Results

### 3.1. Literature Screening Process and Results

**Figure 1:**
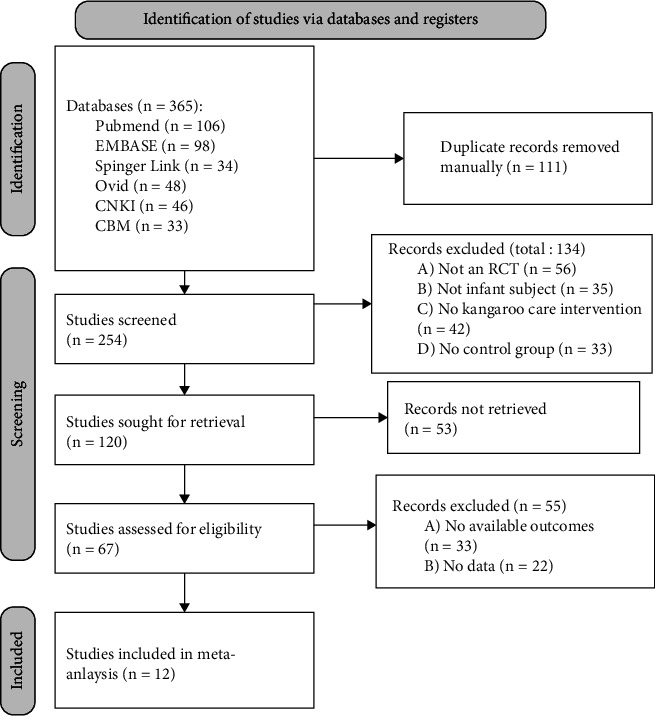
Literature selection flowchart.

Literature identification, screening, and exclusion followed PRISMA's recommendations and are shown in Figure 1. In this study, 365 pieces of literature were initially searched, and these 365 pieces of literature retrieved from the database were deduplicated and screened; only 12 pieces of literature [8, 9, 13–22] were included in the final study.

### 3.2. Basic Characteristics and Patient Characteristics of the Included Pieces of Literature

A total of 1172 infants were included in this study, of whom 585 neonates (49.9%) used KC, 587 neonates (50.1%) used other methods, and 2 literatures (16.7%) were injected with HBV vaccine. In the control group, oral sucrose (or glucose) was used in 6 articles and standard care or minimal care in 6 articles. Basic characteristics of the literature are shown in Table 1.

### 3.3. Literature Bias and Quality Assessment

**Figure 2:**
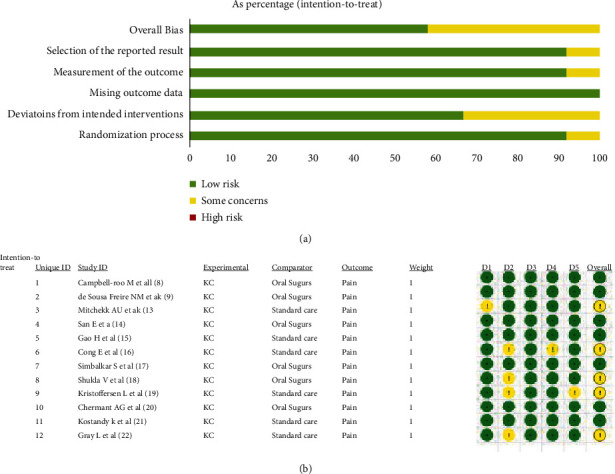
Risk of bias for inclusion of 12 articles based on Cochrane risk of bias 2.0: (a) summary of the bias and (b) detail of the bias.

5 (41.7%) of the 12 articles included in this study [13, 16, 18, 19, 22] had “some risk of bias” in terms of the randomization process, data measurement, intervention deviations, and selective bias, and the remaining 7 (58.3%) articles were “low risk” with a good overall quality. Summary of bias assessed according to Cochrane ROB 2.0 and details of the assessment are summarized in Figures 2(a) and 2(b).

### 3.4. Metaquantitative Analysis Results of Outcome Indicators

#### 3.4.1. PIPP Score after the Procedure

**Figure 3:**
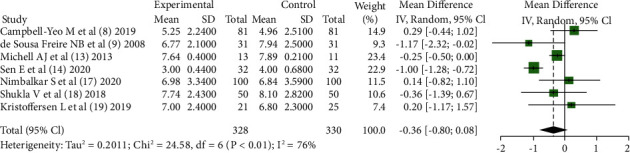
The PIPP score after the procedure: comparing KC with other interventions.

Among the 12 included pieces of literature, 7 pieces of literature [8, 9, 13, 14, 17–19] tried to draw the comparison between KC and other nondrug intervention methods in invasive procedures for infants and reported the PIPP score indicators, 5 pieces of literature [8, 9, 14, 17, 18] were treated with sucrose intervention in the control group, and 2 pieces of literature [13, 19] were treated with standard care in the control group. Combined results showed that 7 articles presented statistical heterogeneity (*Chi*^2^ = 24.58, *df* = 6, *P* < 0.01), and the random-effect model was used to obtain a combined effect size (*MD* = −0.36, 95% CI (−0.80, 0.08), *Z* = −1.60, *P*=0.11), indicating KC compared with other nonpharmacological interventions; there was no clear advantage for pain reduction in infants as shown in Figure 3.

#### 3.4.2. Heart Rate during the Procedure

**Figure 4:**
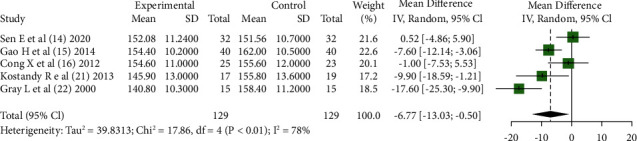
Heart rate during the procedure: comparing KC with other interventions.

5 articles [14–16, 21, 22] reported the comparison of the heart rate between KC and other nonpharmacological interventions in invasive procedures, and the combined results showed that 5 articles were statistically heterogeneous (*Chi*^2^ = 17.86, *df* = 4, *P* < 0.01); the fixed-effect model was used to obtain a combined effect size (*MD* = −6.77, 95% CI (−13.03, −0.50), *Z* = −2.12, *P*=0.03), indicating that infants under KC intervention had significantly smaller heart rates during invasive procedures than those in other interventions as shown in Figure 4.

#### 3.4.3. Heterogeneity Investigation

**Figure 5:**
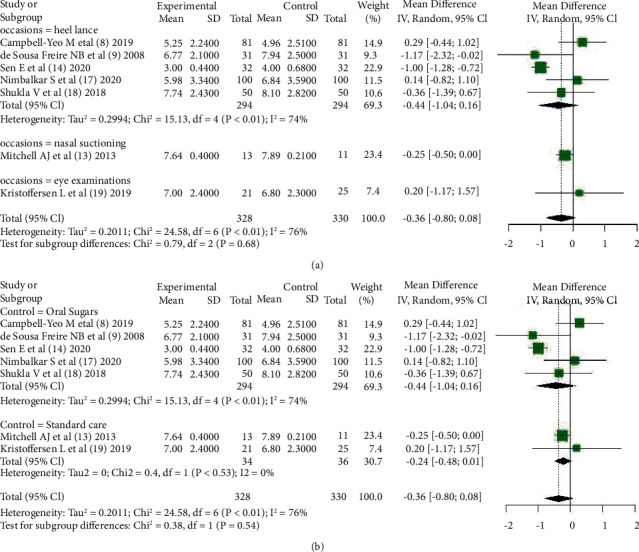
Subgroup analysis according to (a) occasions or (b) control methods.

In the combined analysis of the PIPP score, heterogeneity between articles was statistically significant, and all 7 articles were divided into subgroups according to the scenario of intervention application and according to the way of intervention in the control group. However, heterogeneity between subgroups was not statistically significant (*P*=0.68; *P*=0.54), which illustrated the scenario of intervention application, and the mode of intervention in the control arm was not a source of heterogeneity as shown in Figures 5(a) and 5(b).

#### 3.4.4. Influence Analysis

**Figure 6:**
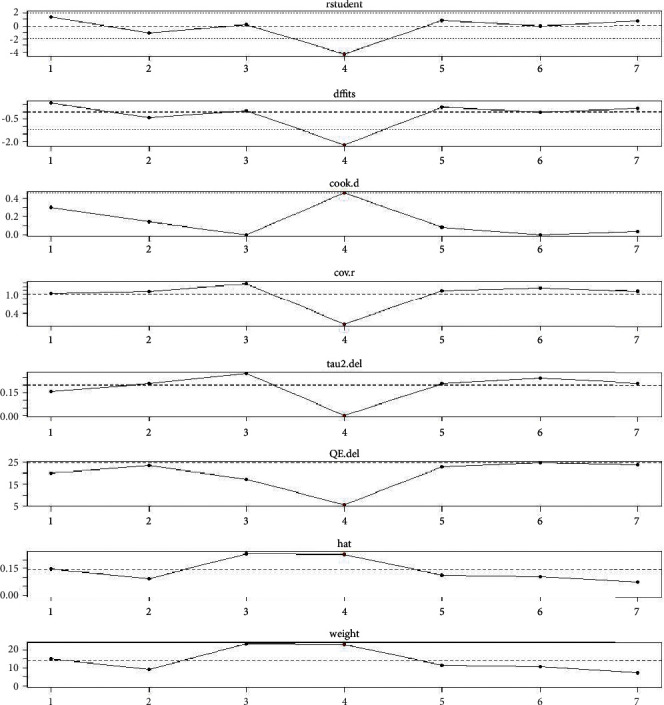
Influence analysis.

In the influence analysis on PIPP score outcome indicators, it was found that all pieces of literature were within the acceptable range, indicating a good stability, as shown in Figure 6.

#### 3.4.5. Publication Bias Analysis

**Figure 7:**
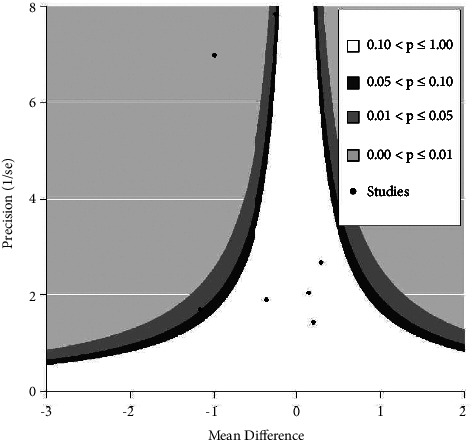
Contour-enhanced funnel plot.

In the combined analysis of PIPP score outcome indicators, publication bias was measured using Egger's test for the results, *t* = 0.66, *P*=0.54, indicating that there was no publication bias, and the contour-enhanced funnel plot is shown in Figure 7.

## 4. Discussion

Although newborns are unable to express pain in words, they have perceptual instinct to negative stimuli such as pain and can show significant physiological responses, such as increased heart rate, increased blood pressure, decreased oxygen saturation, and increased respiratory rate, and neonatal pain can be comprehensively evaluated with the help of behavioral responses such as crying and duration of painful facies[23]. Therefore, the PIPP score, heart rate, and oxygen saturation indexes were selected in this study to comprehensively evaluate the effect of kangaroo care on relieving neonatal pain caused by invasive procedures.

Among the 12 pieces of literature included in this study, only 7 pieces of literature reported the PIPP score, 6 pieces of literature reported the heart rate, and 1 literature reported oxygen saturation. Meta-analysis showed that KC had no significant advantage over other nondrug interventions in the overall assessment of infant pain; however, infants' heart rate during invasive procedures with KC intervention was significantly lower than in other interventions. Because there was only one literature reporting oxygen saturation, a meta-analysis was not performed at that time. Although KC is not more effective in reducing pain than oral sucrose (or glucose) or standard care, it remains positive for neonatal pain relief, which may be due to kangaroo care inhibiting the activity of the hypothalamic-pituitary-adrenocortical axis, reducing salivary cortisol, serum cortisol, and *β*-endorphin secretion levels, and stimulating C-afferent fibers to excite the limbic system of the brain to produce a sense of pleasure and inhibit the conduction of pain signals, thereby reducing operant pain and resulting in a decreased heart rate [24–26]. However, also as a nonpharmacological intervention, oral sucrose (or glucose) is also an important intervention, and sucrose and glucose are the most commonly used sweeteners, which are effective and simple to use, and have no documented side effects and have gained wide use in the clinical setting for management of neonatal pain [27, 28]. In the literature [8], although there was no significant difference in PIPP scores between KC alone and oral sucrose, the investigators combined oral sucrose with KC in 81 neonates, and compared with oral sucrose, PIPP scores in KC were significantly lower than those in the oral sucrose group, which suggests that KC combined with oral sucrose is coapplied in children to reduce pain during invasive procedures, and this may lead to better results. This was mapped in literature [20]; however, as this was not the purpose of this study, it could be left for verification by subsequent studies.

In literature [16], the investigators randomized two experimental groups, in which KC started 30 min or 15 min before neonatal heel blood sampling, and the results showed no difference in pain relief between the two groups, which showed that KC had little effect when it started, and it was important that maternal contact and comfort were provided for neonatal pain stimulation to reduce pain and anxiety. In a study by Shukla et al. [29], comparing the effect of KC performed by fathers and mothers separately, the results showed no significant difference, which may be related to the fact that newborns are not sensitive to the giver of KC.

In this meta-analysis, heterogeneity in pieces of literature was obvious, but we performed subgroup analysis according to factors that may cause heterogeneity of the literature (scenario of intervention application and the mode of intervention in the control group), and there was no significant difference between the groups. This suggested that the source of heterogeneity is not related to the scenario of intervention application, and the mode of intervention in the control group may be related to different characteristics of newborns, different strategies implemented by KC, and confounding factors such as the number of included sample sizes.

Although the quality evaluation by Cochrane ROB 2.0 of 12 articles suggested that 7 articles had “some concerns of risk,” the overall quality of the articles was good, the results were stable, and there was no publication bias. Because different pieces of literature had diversified reporting indicators for pain, for example, some pieces of literature [20] adopted the NFCS score to report pain, and many other studies [30, 31] were excluded because there were no available data, which makes this analysis not comprehensive. For this topic, more studies with better quality still need to be included from different perspectives with different indicators for in-depth analysis. On the other hand, the impact of conventional NSAIDs or antibiotics, e.g., aspirin [32], amoxicillin [33], etc., on neonatal health also deserves attention.

The heart rate of KC intervention during invasive procedures in infants was significantly lower than that of other interventions, significantly relieving pain in infants, but the effect did not exceed that of oral sucrose (or glucose) or standard care, and the combination of KC and oral sucrose may have a better effect on pain relief in infants, but more studies are still needed to verify this effect.

## Figures and Tables

**Table 1:** Basic characteristics, patient characteristics, and outcome indicators of the included pieces of literature.

Author	Year	Intention-to-treat total	Number of the experimental group	Number of the control group	GA (weeks)	Painful stimulus occasions	Experimental group method	Control group method	Outcome indicators
Campbell-Yeo et al. [8]	2019	162	81	81	32.6 ± 2.1	Heel lance	KC through the procedure	Oral Sucrose (1 ml, 24%)	(a)

de Sousa Freire et al. [9]	2008	62	31	31	33.7 ± 1.57	Heel lance	KC through the procedure	Oral glucose (1 ml, 25%)	(a)

Mitchell et al. [13]	2013	26	13	11	29	Nasal suctioning	2 hours daily skin-to-skin holding	Minimal parent holding	(a)

Sen et al. [14]	2020	64	32	32	34.38 ± 1.59	Heel lance	KC through the procedure	Oral Sucrose (0.5 ml, 24%)	(a) (b) (c)

Gao et al. [15]	2014	80	40	40	37	Heel lance	KC through the procedure	Standard care	(b)

Cong et al. [16]	2012	48	25	23	30 ± 2.2	Heel lance	KC through the procedure	Standard care	(b)

Nimbalkar et al. [17]	2020	200	100	100	33.6 ± 1.89	Heel lance	KC through the procedure	Oral Sucrose (1 ml, 24%)	(a) (c)

Shukla et al. [18]	2018	100	50	50	32.79 ± 2.34	Heel lance	KC through the procedure	Oral Sucrose (1 ml, 24%)	(a) (c)

Kristoffersen et al. [19]	2019	46	21	25	33 ± 2	Eye examinations	KC through the procedure	Standard care	(a)

Chermont et al. [20]	2009	320	160	160	39 ± 1	Intramuscular injection of hepatitis B vaccine	KC through the procedure	Oral glucose (1 ml, 25%)	(d)

Kostandy et al. [21]	2013	36	17	19	39.7 ± 1.29	Intramuscular injection of hepatitis B vaccine	KC through the procedure	Standard care	(b)

Gray et al [22]	2000	30	15	15	NA	Heel lance	KC through the procedure	Standard care	(b)

*Note.* PIPP, premature infant pain profile; GA, gestational age; KC, kangaroo care; HR, heart rate; SPO_2_, pulse oxygen saturation; NFCS, neonatal facial coding system; and NA, not available. Outcomes: (a) PIPP score after the procedure; (b) HR value during the procedure; (c) SPO_2_ during the procedure; (d) the NFCS Score.

## Data Availability

The data used in this study are available from the corresponding author upon request.

## References

[1] Koller D., Goldman R. D. (2012). Distraction techniques for children undergoing procedures: a critical review of pediatric research. *Journal of Pediatric Nursing*.

[2] Pancekauskaitė G., Jankauskaitė L. (2018). Paediatric pain medicine: pain differences, recognition and coping acute procedural pain in paediatric emergency room. *Medicina (Kaunas)*.

[3] Harrison D., Reszel J., Bueno M. (2016). Breastfeeding for procedural pain in infants beyond the neonatal period. *Cochrane Database of Systematic Reviews*.

[4] Stevens B., Johnston C., Petryshen P., Taddio A. (1996). Premature infant pain profile: development and initial validation. *The Clinical Journal of Pain*.

[5] Filippa M., Monaci M. G., Spagnuolo C., Serravalle P., Daniele R., Grandjean D. (2021). Maternal speech decreases pain scores and increases oxytocin levels in preterm infants during painful procedures. *Scientific Reports*.

[6] McNair C., Campbell-Yeo M., Johnston C., Taddio A. (2019). Nonpharmacologic management of pain during common needle puncture procedures in infants: current research evidence and practical considerations: an update. *Clinics in Perinatology*.

[7] Zengin H., Cinar N. (2022). Designing dress (Sarbebe) for kangaroo care, the effect of kangaroo care provided with this dress on mother and newborn’s comfort. *Health Care for Women International*.

[8] Campbell-Yeo M., Johnston C. C., Benoit B. (2019). Sustained efficacy of kangaroo care for repeated painful procedures over neonatal intensive care unit hospitalization: a single-blind randomized controlled trial. *Pain*.

[9] de Sousa Freire N. B., Garcia J. B. S., Lamy Z. C. (2008). Evaluation of analgesic effect of skin-to-skin contact compared to oral glucose in preterm neonates. *Pain*.

[10] Stevens B., Yamada J., Ohlsson A. (2004). Sucrose for analgesia in newborn infants undergoing painful procedures. *Cochrane Database of Systematic Reviews*.

[11] Minozzi S., Dwan K., Borrelli F., Filippini G. (2022). Reliability of the revised Cochrane risk-of-bias tool for randomised trials (RoB2) improved with the use of implementation instruction. *Journal of Clinical Epidemiology*.

[12] Stevens B. J., Gibbins S., Yamada J. (2014). The premature infant pain profile-revised (PIPP-R): initial validation and feasibility. *The Clinical Journal of Pain*.

[13] Mitchell A. J., Yates C. C., Williams D. K., Chang J., Hall R. W. (2013). Does daily kangaroo care provide sustained pain and stress relief in preterm infants?. *Journal of Neonatal-Perinatal Medicine*.

[14] Sen E., Manav G. (2020). Effect of kangaroo care and oral sucrose on pain in premature infants: a randomized controlled trial. *Pain Management Nursing*.

[15] Gao H., Xu G., Gao H. (2015). Effect of repeated Kangaroo Mother Care on repeated procedural pain in preterm infants: a randomized controlled trial. *International Journal of Nursing Studies*.

[16] Cong X., Cusson R. M., Walsh S., Hussain N., Ludington-Hoe S. M., Zhang D. (2012). Effects of skin-to-skin contact on autonomic pain responses in preterm infants. *The Journal of Pain*.

[17] Nimbalkar S., Shukla V. V., Chauhan V. (2020). Blinded randomized crossover trial: skin-to-skin care vs. sucrose for preterm neonatal pain. *Journal of Perinatology*.

[18] Shukla V., Chapla A., Uperiya J., Nimbalkar A., Phatak A., Nimbalkar S. (2018). Sucrose vs. skin to skin care for preterm neonatal pain control-a randomized control trial. *Journal of Perinatology*.

[19] Kristoffersen L., Støen R., Bergseng H. (2019). Skin-to-skin contact during eye examination did not reduce pain compared to standard care with parental support in preterm infants. *Acta Paediatrica*.

[20] Chermont A. G., Falca˜o L. F. M., de Souza Silva E. H. L., de Cássia Xavier Balda R., Guinsburg R. (2009). Skin-to-skin contact and/or oral 25% dextrose for procedural pain relief for term newborn infants. *Pediatrics*.

[21] Kostandy R., Anderson G. C., Good M. (2013). Skin-to-skin contact diminishes pain from hepatitis B vaccine injection in healthy full-term neonates. *Neonatal Network*.

[22] Gray L., Watt L., Blass E. M. (2000). Skin-to-skin contact is analgesic in healthy newborns. *Pediatrics*.

[23] Cong X., Ludington-Hoe S. M., McCain G., Fu P. (2009). Kangaroo Care modifies preterm infant heart rate variability in response to heel stick pain: pilot study. *Early Human Development*.

[24] Ludington-Hoe S. M., Hosseini R., Torowicz D. L. (2005). Skin-to-skin contact (Kangaroo Care) analgesia for preterm infant heel stick. *AACN Clinical Issues: Advanced Practice in Acute and Critical Care*.

[25] Castral T. C., Warnock F., Leite A. M., Haas V. J., Scochi C. G. (2008). The effects of skin-to-skin contact during acute pain in preterm newborns. *European Journal of Pain*.

[26] Okan F., Ozdil A., Bulbul A., Yapici Z., Nuhoglu A. (2010). Analgesic effects of skin-to-skin contact and breastfeeding in procedural pain in healthy term neonates. *Annals of Tropical Paediatrics*.

[27] Liaw J. J., Yang L., Katherine Wang K. W., Chen C. M., Chang Y. C., Yin T. (2012). Non-nutritive sucking and facilitated tucking relieve preterm infant pain during heel-stick procedures: a prospective, randomised controlled crossover trial. *International Journal of Nursing Studies*.

[28] Pillai Riddell R. R., Racine N. M., Gennis H. G. (2015). Non-pharmacological management of infant and young child procedural pain. *Cochrane Database of Systematic Reviews*.

[29] Shukla V. V., Chaudhari A. J., Nimbalkar S. M., Phatak A. G., Patel D. V., Nimbalkar A. S. (2021). Skin-to-Skin care by mother vs. Father for preterm neonatal pain: a randomized control trial (environ trial). *International Journal of Pediatrics*.

[30] Patel D. V., Soni S. N., Shukla V. V. (2022). Efficacy of skin-to-skin care versus swaddling for pain control associated with vitamin K administration in full-term neonates: a randomized controlled trial. *Journal of Tropical Pediatrics*.

[31] Kashaninia Z., Sajedi F., Rahgozar M., Noghabi F. A. (2008). The effect of Kangaroo Care on behavioral responses to pain of an intramuscular injection in neonates. *Journal for Specialists in Pediatric Nursing*.

[32] Cheng X., Huang F., Zhang K., Yuan X., Song C. (2018). Effects of none-steroidal anti-inflammatory and antibiotic drugs on the oral immune system and oral microbial composition in rats. *Biochemical and Biophysical Research Communications*.

[33] Cheng X., He F., Si M. (2022). Effects of antibiotic use on saliva antibody content and oral microbiota in Sprague Dawley rats[J]. *Frontiers in Cellular and Infection Microbiology*.

